# Challenges and facilitators of professional socialization: A systematic review

**DOI:** 10.1002/nop2.341

**Published:** 2019-07-16

**Authors:** Waliu Jawula Salisu, Nahid Dehghan Nayeri, Ibrahim Yakubu, Fatemeh Ebrahimpour

**Affiliations:** ^1^ School of Nursing and Midwifery Tehran University of Medical Sciences Tehran Iran; ^2^ Nursing and Midwifery Training College Gushegu Ghana

**Keywords:** nurses, nursing, profession, socialization, systematic review, undergraduate

## Abstract

**Aims:**

This current study aimed to present a review of the challenges and facilitators of professional socialization among undergraduate nursing students.

**Design:**

Systematic review.

**Methods:**

A literature search was conducted in Embase, Google Scholar, PubMed and Scopus in April and May 2018 for studies published in the English language. Four thousand three hundred fifty‐two articles were retrieved. We conducted further screening for full‐text articles after discarding duplicates and irrelevant studies. Finally, eight studies were included. The Joanna Briggs appraisal tools were used to appraise and evaluate study quality. The PRISMA guidelines were followed and a narrative synthesis used for data analysis.

**Results:**

Challenges and facilitators of professional socialization were identified and categorized into two major themes. Under each theme, results were grouped into three sub‐themes: professional, personal and educational challenges/facilitators.

**What does this paper contribute to the wider global clinical community?**

- The current review presents evidence of existing challenges during the process of professional socialization among undergraduate nursing students.
- The facilitators identified in this review could guide and serve as a yardstick to nursing professionals who would wish to promote socialization.
- Individual efforts are needed by enthusiastic students who aim to achieve appreciable levels of socialization in the profession. However, the contribution of academic and clinical mentors remains significant.

## 1 INTRODUCTION

Professional socialization, henceforth referred to as socialization in this study, involves novices of a professional group getting immersed in the professional culture. It is a process that begins with getting acquainted with the professional roles and gaining a professional identity. Socialization is unavoidable in venturing into any professional body (Moradi et al., 2017). Consciously or otherwise, a profession, through its experienced members, instils the professional attitudes and values in neophytes. Howkins & Ewens (1999) defined socialization as ‘a process by which professionals learn during their education and training, the values, behaviors and attitudes necessary to assume their professional role’. Dinmohammadi, Peyrovi, & Mehrdad (2013) proposed that socialization in the context of nursing be defined as ‘a dynamic, interactive process through which attitudes, knowledge, skills, values, norms and behaviors of the nursing profession are internalized and professional identity is developed’.

## 2 BACKGROUND

Socialization introduces students to hidden cultures, practices and values of the profession, to develop professionalism (Black, 2014). Thus, they may not have the opportunity to develop after graduation and during the early days of practice in the job setting (Bisholt, 2012). Nursing and other health professionals go through the process of socialization to acquire professional skills, values, attitudes, knowledge and other ways of life established in the professional subculture through social interaction with experts and experienced personnel in the professional group (Ryynänen, 2001). The first of many steps in socializing student nurses into professional practice is first to be a nursing student (Black, 2014) since socialization is an extensive process from being a student till a qualified practicing nurse and beyond. Socialization could be formal (to include classroom lectures, seminars, undertaking assignments, arranged meetings with a mentor) or informal (unplanned observations, participation in student associations) (Black, 2014). Whichever form it takes, it provides constant psychological readiness to students for future practice, with the opportunity for new roles and attitudes to be learned. As individuals acquire new roles, they are automatically recognized and accepted into newer social groups (Shinyashiki, Mendes, Trevizan, & Day, 2006).

Undergraduate nursing students require mentoring through socialization by experienced leaders in the nursing profession, which often is a mutual understanding and agreement between the mentee and the mentor (Hawkins & Fontenot, 2010). Students achieve essential clinical skills, by moving from one clinical role to another (Tradewell, 1996) and gain competencies through interactive learning with mentors and preceptors which leads to better clinical performance (Shin, Sok, Hyun, & Kim, 2014).

For students to be adequately socialized, they should spend enough time with their mentors, preceptors, role models or qualified senior nurses in the practice settings to gain enough exposure to nursing culture. Communication skills and ethics could as well be integrated into clinical practice to get students adequately socialized (Ryynänen, 2001). The experience gained from experienced practitioners is an essential determinant of socialization (Black, 2014; Nesler, Hanner, Melburg, & McGowan, 2001). Undergraduate nursing students, after successful completion, join the nursing workforce. This transitional process could be a shock to most newly qualified nurses since they face unexpected and challenging job demands (Bisholt, 2012). As students become professional nurses, they will go through an inevitable process of socialization in practice. The freshly graduated nurses will need supervision to function effectively and to be able to adapt to the scientific environment (Bisholt, 2012). The educational process of nursing students goes beyond scientific knowledge acquisition and clinical skills (Shinyashiki et al., 2006). Through socialization, they learn the culture, values, attitudes and behaviours of the nursing profession, which will guide them later in their careers. After all, socialization is a lifelong process and does not end when students graduate and leave school (Black, 2014). Socialization among undergraduate nursing students is a vital component in preparing clinically competent students (Shin et al., 2014) and also in equipping young nurses with research skills, improving their critical thinking and reasoning abilities (Bell, Rominski, Bam, Donkor, & Lori, 2013; Shipman, Roa, & Hooten, 2011). Socialization also prepares students for future leadership positions as they progress to become registered nurses (Bell et al., 2013). Nurses require relevant and updated nursing skills and knowledge to contribute meaningfully to the provision of quality health care (Baldwin, Bentley, Langtree, & Mills, 2014). Studies suggest that higher nursing education has a positive relation with patient outcomes (Shipman et al., 2011). However, this would require socialization to enable nurses to attain a more profound understanding of their profession. The process of socialization among nursing students is sometimes interrupted during the training period, leading to a failed or halted process (Moradi et al., 2017). Several barriers account for this; however, due to the paucity of literature, it remains unclear. Only a few studies are known to focus on how the socialization process among undergraduate nursing students’ trends.

This study aims to present a review of the challenges and facilitators of socialization among undergraduate nursing students. The review will attempt to answer the question; what are the challenges/barriers and facilitators of socialization among undergraduate nursing students? The results will be necessary for identifying relevant issues among undergraduate nursing students both at the clinical and educational setting in their attempt to socialize in the nursing profession.

## 3 DESIGN

Systematic review.

## 4 METHODS

We conducted a systematic review of recent challenges and facilitators of socialization among undergraduate nursing students. Evidence was reported according to the reporting checklist of the Preferred Reporting Items for Systematic Reviews and Meta‐Analysis (PRISMA) statement by the Centre for Reviews and Dissemination (Liberati et al., 2009), see Supplementary File 1. To ensure rigour, we outlined an explicit inclusion and exclusion criteria and only included data from the literature that met the inclusion criteria. Also, potential studies were frequently screened and cross‐checked between authors before inclusion.

### 4.1 Search strategy

The authors conducted a literature search between April–May 2018 in electronic databases such as PubMed, Embase, Google Scholar and Scopus for peer‐reviewed articles published in the English language. Included studies were limited to publications not later than 2008 since we aimed to discover current trends; we believe older studies would be of less relevance to our current study.

Search terms used were ‘socialization’, ‘resocialization’, ‘undergraduate’, ‘nurses’, ‘Bachelor of science’, ‘Education’.

The search strategy used in PubMed advanced search was as follows:

1. Socialization
2. Resocialization
3. 1 Or 2
4. Undergraduate
5. ‘Bachelor of science’.
6. Education
7. Nurse$
8. 4 OR 5 OR 6 OR 7
9. 3 AND 8

For the inclusion of studies, two authors independently resolved issues of discrepancies after assessing the eligibility of literature.

### 4.2 Study selection and data extraction

Studies were imported into an Endnote library by one of the authors (WJS). Duplicates were removed using the ‘find duplicates’ feature of the Endnote software. Two authors (WJS and FE) then screened titles and abstracts of the remaining studies independently and removed all irrelevant studies. Full text of remainder studies was retrieved for further screening and data collection process. From this stage, the full texts were carefully read by three of the authors (WJS, FE and YI) and independently included all available studies based on the inclusion criteria. Where there was a disagreement among authors, a neutral author (NDN) was consulted for consensus. Data extraction was completed using a standard pre‐designed data extraction form in Microsoft Excel spreadsheet by two authors, while the third author provided intellectual guidance. The data extraction form included the following: study (first author's name and date of publication), the country a study was conducted, method, type of participants (population) and sample size (Table 1).

**Table 1:** Characteristics of included studies

Study	Country	Method	Population	Sample size
Holley and Taylor (2009)	USA	Qualitative study	Online undergraduate nursing students	19
Condon and Sharts‐Hopko (2010)	Japan	Qualitative study	Undergraduate nursing students and faculty members	10
Love (2010)	USA	Qualitative study	Undergraduate nursing students	8
Brown et al. (2012)	Australia	Mixed method	Undergraduate nursing students and clinical teachers	14
Curtis et al. (2012)	UK	Qualitative study	Undergraduate nursing students and nurse teacher	24
Zarshenas et al. (2014)	Iran	Qualitative study	Undergraduate nursing students and registered nurses	43
Thomas et al. (2015)	UK	Qualitative study	Undergraduate nursing students	26
Dinmohammadi et al. (2017)	Iran	Qualitative study	Undergraduate/graduate nursing students	14

For the inclusion criteria, studies should have assessed socialization among undergraduate nursing students. Study designs should have been cross‐sectional, mixed method, qualitative or systematic reviews conducted from the year 2008–2018 with clearly defined methods of data collection. We excluded studies that were conducted outside the defined year limits and among other nursing training group aside undergraduate nursing trainees.

### 4.3 Quality appraisal

The final studies that met the inclusion criteria to be considered in this review were independently assessed for quality by two authors (WJS and YI) and crossed checked by another (NDN) before the data analysis. The Joanna Briggs (JBI, 2017) appraisal tool was used to appraise and certify studies for inclusion and exclusion. The tool is made of a checklist comprising 10 questions for qualitative studies, eight for cross‐sectional studies and 11 for systematic reviews and research syntheses. All the outlined studies met the inclusion standard of the appraisal tools before they were considered. If there were any disagreements, it was resolved through discussions until a consensus was reached.

### 4.4 Synthesis of results

A narrative synthesis was used to conduct the data analysis. This method allows a combination of qualitative and quantitative evidence to be summarized together using a textual approach (Pope, Mays, & Popay, 2007). We extracted and grouped the results of interest from the included studies. These were labelled as major findings. Results that answered the review question were those of interest; therefore, those were what we included. Essential statements from the major findings that represent current debates in socialization were noted down as our evidence and further categorized into two major themes—challenges and facilitators. Under each theme, results were discussed under the following sub‐themes: professional factors, personal factors and educational factors.

Research Ethics Committee approval was not required for this current study.

## 5 RESULTS

### 5.1 Study selection

**Figure 1:**
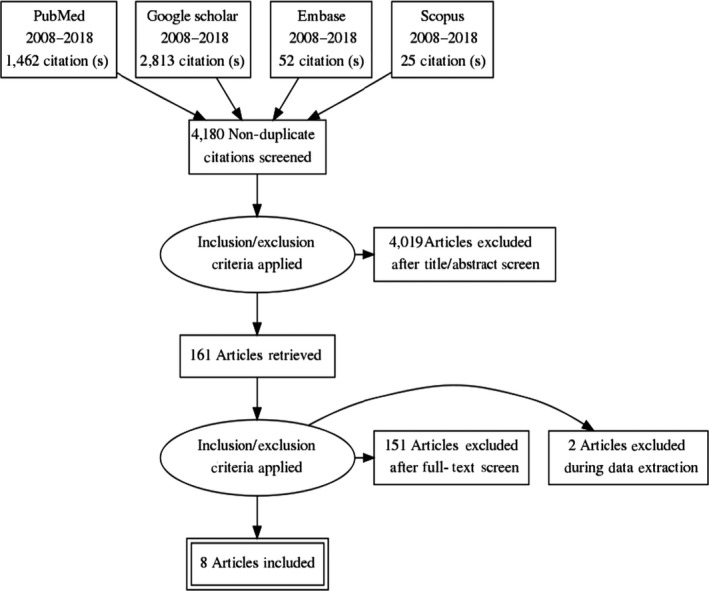
Flow diagram of the literature search

Out literature search yielded 4,352 articles. Using endnote software, 172 articles were discarded as duplicates. After the primary screening of titles and abstracts, 4,019 articles were further discarded for being irrelevant to the current study. The secondary screening was conducted for 161 full‐text articles. Two articles were excluded because we could not have access to the full text and efforts to reach the corresponding authors were not successful. Another 151 articles were excluded; among these, 117 focused on professional nurses/doctors alone without students, 22 were conducted among post‐graduate and Ph.D. level nurses, and 12 were review articles that did not focus entirely on undergraduate nursing students. In the end, eight studies met the inclusion criteria and were included in the review. Figure 1 shows a flow diagram of the literature search.

### 5.2 Characteristics of included studies

All eight included studies were published between 2008–2017 (Brown, Stevens, & Kermode, 2012; Condon & Sharts‐Hopko, 2010; Curtis, Horton, & Smith, 2012; Dinmohammadi, Peyrovi, & Mehrdad, 2017; Holley & Taylor, 2009; Love, 2010; Thomas, Jinks, & Jack, 2015; Zarshenas et al., 2014) the sample size ranged from 14–43 with a total population of at least 144 participants. Table 1 contains the complete characteristics of the included studies.

In line with the aims of this review, we identified and extracted the challenges and facilitators of socialization among undergraduate nursing students from the included studies as illustrated in Table 2.

**Table 2:** Challenges and facilitators of professional socialization

First author and year of publication	Challenges of professional socialization			Facilitators of professional socialization		
	Professional challenges	Educational challenges	Individual challenges	Professional facilitators	Educational facilitators	Individual facilitators
Holley and Taylor (2009)		Independent learning programmes, limited interactions			Interactive learning, academic support	Social interaction, desire to seek professional knowledge
Condon and Sharts‐Hopko (2010)		Focused education		Team Building, working with Peers	Extracurricular networking	Working with peers, openness to others, communication, reflection, role observation
Love (2010)	Discrimination and isolation					Learning with friends, the strength to pursue more, pressure to succeed, fitting in
Brown et al. (2012)					Supportive clinical teachers and mentors	
Curtis et al. (2012)	Dissonance between professional ideals and practice reality		Time limitation	Realignment between the reality of practice and professional ideals, leadership to align practice reality with professional ideals, collaboration	Fostering resilience to maintain professional ideals, greater education, supportive teachers	Maintaining balance
Zarshenas et al. (2014)	Theory‐practice incongruence and tacit knowledge		Personal beliefs	Professional support, relatedness		Internal motivation, forming professional identity, Sense of belonging, educational experiences
Thomas et al. (2015)						Status negotiation, status relocation
Dinmohammadi et al. (2017)	Integration, extra‐professional context, extra‐professional agents and conditions		Dependence, disintegration	Professional context, professional agents and conditions		

#### 5.2.1 Challenges of Socialization

##### Professional factors

Under this subcategory, we identified dissonance between professional ideals and practice reality (Curtis et al., 2012), integration, extra‐professional context, extra‐professional agents and conditions (Dinmohammadi et al., 2017), discrimination and isolation (Love, 2010) and also theory–practice incongruence and tacit knowledge (Zarshenas et al., 2014).

##### Personal factors

Time limitation (Curtis et al., 2012), dependence, disintegration (Dinmohammadi et al., 2017) and personal beliefs (Zarshenas et al., 2014).

##### Educational factors

Focused education (Condon & Sharts‐Hopko, 2010), independent learning programmes and limited interactions (Holley & Taylor, 2009).

#### 5.2.2 Facilitators of Socialization

##### Professional factors

Team building, working with peers (Condon & Sharts‐Hopko, 2010), realignment between the reality of practice and professional ideals, leadership to align practice reality with professional ideals, collaboration (Curtis et al., 2012), professional context, professional agents and conditions (Dinmohammadi et al., 2017), professional support and relatedness (Zarshenas et al., 2014).

##### Personal factors

Openness to others, communication, reflection, role observation (Condon & Sharts‐Hopko, 2010), maintaining balance (Curtis et al., 2012), learning with friends, the strength to pursue more, pressure to succeed, fitting in (Love, 2010), status negotiation, status relocation (Thomas et al., 2015), social interaction, desire to seek professional knowledge (Holley & Taylor, 2009), internal motivation, forming professional identity, sense of belonging and educational experiences (Zarshenas et al., 2014).

##### Educational factors

Extracurricular networking (Condon & Sharts‐Hopko, 2010), fostering resilience to maintain professional ideals, greater education, supportive teachers (Curtis et al., 2012), supportive clinical teachers and mentors (Brown et al., 2012), interactive learning and academic support (Holley & Taylor, 2009).

## 6 DISCUSSION

The current review presented herein highlights recent challenges and facilitators of socialization among undergraduate nursing students. After successful completion of a nursing programme, undergraduate nursing students undergo a transition from graduate nurses to registered nurses (Zinsmeister & Schafer, 2009). During this period, experiences gained through socialization in the course of their training could either be supportive or an impediment towards their future career. Nurse's ability to successfully undergo the transition process will depend on whether they were adequately socialized or not. Nurses who could not achieve adequate socialization during their training may face challenges, especially during the early days of work. As posited by Zinsmeister and Schafer (2009), supportive work environment, positive preceptor experience, clarity of role expectations, comprehensive orientation process, sense of professionalism and self‐confidence are supportive agents in the transition process.

For socialization to occur, there must be a profession with members who are keen to be recognized in it. Despite the enthusiasm, the members may encounter setbacks in their attempt to socialize in the profession thoroughly. These may arise due to the professional set‐up and structures or interpersonal glitches among members of the profession. For example, we found that nursing students face career‐related challenges such as discrimination, disrespect and being isolated by other members of the nursing profession during training. In circumstances such as this, students become withdrawn and lose interest in the training process when they actually could develop a positive self‐image and professional identities through interacting with other members of the profession. Such situations could have a long‐term effect on the nurse–patient relationship of such nurses since other studies suggest that positive self‐image is essential for nurses to develop stronger therapeutic relationships with patients (Öhlén & Segesten, 1998). Also, since professional identity is acquired through socialization and develops throughout nurses’ training period, it evolves during career years helping to build confidence, self‐esteem and autonomy of nurses (Johnson, Cowin, Wilson, & Young, 2012; Seo & Kim, 2017). Even though some studies agree socialization plays a vital role in the development of self‐esteem (Randle, 2003), Mooney suggests nurses could have low self‐esteem and lack assertiveness due to how socialization is implemented (Mooney, 2007).

An effective socialization process relies on effective knowledge transfer and practice to boost the continuity and maintenance of professional values. Students face practical realities in clinical settings and are required to apply their theoretical knowledge in practice. The dissonance between professional ideals and practical reality has an impact on students, making them feel vulnerable and attempting to maintain a balance. As neophytes, this often becomes burdensome since they lack the necessary experience and expertise. Unable to meet and deal with this demand, students would usually feel less of themselves, withdrawn and demotivated. Others may consider themselves unfit and sometimes lack confidence and the desire to freely interact with peers and clinical teachers—halting the socialization process. This is congruent with findings of a study by Cheraghi and colleagues; they reported that student's learning experiences in the academic settings were unlike the skill demands of the clinical setting, making the clinical environment unconducive for students (Cheraghi, Salasli, & Ahmadi, 2007).

According to the findings of this study, students frequently suffer a state of disintegration where they lack appropriate clinical knowledge to function effectively. This situation exposes students to distress and uncertainty. Findings of a similar study by Swardt, Rensburg, and Oosthuizen (2017) presented guidelines to support professional nurses and educators in the socialization of students. They reported in their study that students experienced a lack of support and witnessed incivility in the clinical environment. These circumstances expose students to feelings of timidity and attitudes of withdrawal. On the continuum of socialization, both professionals and students benefit from professional support to maintain consistent growth. When this is lacking, it leads to adverse outcomes, which has the potential to halt or disrupt the socialization process. Previous studies identified accidental socialization (Swardt et al., 2017), demoralization, lowered productivity, frequent displacements, negative body image and non‐acceptance of roles as possible adverse outcomes of a failed socialization process (Moradi et al., 2017). Also, Mooney (2007) reported other consequences of a lack of professional support. Mooney posited that newly qualified nurses become discouraged when they attempt to be vocal and do not feel protected by their senior colleagues.

Students encounter personal and interpersonal problems, and depending on the magnitude, their socialization process could be faced with challenges. For example, we found that personal beliefs and preconceived ideas held by students about nursing affect their attitude towards socialization. Academic and clinical supervisors must collaborate to curtail unjustified beliefs as soon as students begin their study programmes rather than allow them to linger on it. Correspondingly, Secrest and colleagues suggested in their study that reflective courses and seminars on professionalism should not be delayed. Instead, students should be exposed to aspects of professionalism early enough during their training (Secrest, Norwood, & Keatley, 2003). By this, students will resolve any preconceived ideas and beliefs held by students early enough to allow a smooth transition and commencement of the socialization process. There is also a chance for students to develop positive attitudes towards the chosen profession. Congruent to this, Zamanzadeh, Roshangar, Fathi‐Azar, Valizadeh, and Kirkwood (2014) in their study, which examined the strategies used by newly graduated Iranian nurses to gain self‐confidence during their early professional career, reported that orientation and training sessions are held for new graduates. These programmes serve to welcome the graduates and keep them abreast with the new environment, eventually, becoming a source of motivation and a way to develop self‐confidence. In the long run, early commencement of programmes that aim to clarify professional roles and expectations for students will help to clear misconceptions and serve to facilitate the socialization process.

In this study, our findings suggest that the type of educational programme that students enrol in during their training could influence their socialization process. For example, independent learning programmes such as structured online curricular leads to students having limited interactions with peers and faculty. A point that buttresses the fact that student's communication and informal interactions, which are easy ways to socialize, is minimal in these programmes. It is mainly because those programmes are often individually centred and web‐based, without the opportunity for students to meet physically. Similarly, we found that some nursing programmes are structured to focus on specific curricular without room for diversity. This often confines students’ perspectives with minimal opportunity for open‐mindedness. These together result in limited chances for students to socialize adequately. In as much as clinical experiences and interactions among and between students and faculty play an essential role in the socialization process, Nesler et al. (2001) argue differently. In their study, they suggest that students enrolled in distant nursing programmes acquire comparable socialization outcomes to those enrolled in on‐campus programmes (Nesler et al., 2001). The critical aspect of it could be to create the student's indulgence and preach to them the attitudes, values, cultures and knowledge of the nursing profession.

Socialization benefits nurses and other professionals in ways such as the acquisition of professional skills and identity, easy adaptability to new professional roles and environment, which could lead to an improvement in the quality of care (Dinmohammadi et al., 2013). However, a student's ability to form professional identity rests on the socialization process they have been exposed to (Moradi et al., 2017). Studies suggest socialization allows students to acquire self‐identification and opportunities to learn the educational content of the nursing profession, which often leads to internalization of values and goals associated with the profession (Simpson, 1967). Even though socialization involves the transmission of knowledge and professional skills, its role transcends this. Socialization assists nurses in developing self‐awareness and nursing consciousness. These are necessary for guiding personal feelings, beliefs and values against interfering in the provision of nursing care (Black, 2014).

Regarding professional facilitators, we found that as neophytes, student nurses require professional guidance and support to enable them to function and adapt appropriately. This may often reflect as supportive teachers and mentors, providing the necessary professional guidance to students as found in this study. Often, experienced nurse educators and clinical teachers offer professional support through interactions with students. A situation creates a relaxed atmosphere for socialization to flourish. This responsibility should not be limited to educators and clinical teachers. In tandem, Swardt et al. (2017) suggest that nursing educational institutions equally have a role to play in providing support to nursing students. Once this supportive relationship exists, it creates a sense of motivation and improves job satisfaction (Swardt et al., 2017), which has a strong relationship with socialization. This is consistent with the results of a study by Mbambo (2014), which assessed the relationship between socialization and job satisfaction of nurse educators. Their findings revealed a positive relationship between socialization and job satisfaction, demonstrating the importance of job satisfaction in facilitating the socialization process (Mbambo, 2014).

We found that teachers promote socialization among students by creating a conducive and supportive environment and by not expecting so much or burdening students with unachievable goals. This allows students to adjust to the realities of practice and to deal with dissonance. This finding is consistent with many other studies (Bartlett, Lucy, Bisbee, & Conti‐Becker, 2009; Byszewski, Gill, & Lochnan, 2015; Hawkins & Fontenot, 2010; Ousey, 2009). As new members of the profession, students will soon face the realities of both the professional practice and ideals, exposing them to experiences of dissonance, leading to uncertainty and a feeling of vulnerability. Curtis et al. (2012) suggest that clinical and academic instructors should collaborate to identify this challenge in compassionate practice, as that could aid student's socialization process. Nursing leadership at the forefront of significant decision‐making positions will as well need to collaborate to identify challenges of dissonance between nursing practice realities and professional ideals, as these challenges usually go beyond studentships to affect newly qualified and early career nurses in the practice setting.

As students encounter different personalities and situations during their training, we found that these were categorized into professional agents and conditions. The professional agents, human factors, and professional conditions, nonhuman factors, are said to influence the socialization process of students positively, thus facilitating the process of socialization or, otherwise, inhibiting the process. In similar studies, human factors, such as mentors, have been described by Ousey (2009) as central to student learning. Their study concludes that mentors would be better positioned to facilitate adequate socialization of students if they are knowledgeable and enthusiastic about their jobs.

Personal characteristics such as us, being opened and ready to communicate freely with colleagues were found in this study as personal facilitators which enable socialization. To individuals with such personal traits, it becomes a source of motivation and a way to learn and discover the hidden cultures and values of the profession they find themselves. Openness further aids students to develop a sense of belongingness and formation of professional identity. Excellent communication promotes interpersonal development and boosts confidence between students and supervisors. In their study, Seo & Kim examined the professional identity of immigrant nurse practitioners and found that patient‐centred thinking formed an essential component of professional identity since it enhanced communication (Seo & Kim, 2017).

Similarly, our findings suggest that other personal characteristics such as students who are opened to collaborations, readiness to work with peers towards common goals and having frequent interactions facilitate socialization. Increased interactions, information sharing and collaborating as teams to collectively solve problems result in a more positive impact on patients than individual effort. Congruent to other studies, Dickerson and Latina (2017) posited that team nursing improves staff satisfaction and patient safety. Also, improved communication and sharing of responsibilities and skills are enhanced through team nursing.

Another important finding of this study is that students were noted to rely on strategies such as negotiation and relocation during the socialization process to fill gaps in their learning needs, moreover, also, to increase their learning experiences, and to relocate or maintain studentship positions and values. Students schedule meetings with other health professionals who are devoted to guide and support them to achieve personal learning goals. During the process, students relocate their status and accept current situations to allow for a successful and easy socialization process. This finding is similar to findings of a study conducted by Perna and Hudgins (1996) which reported that students who had informal meetings and frequently interacted with faculty were more productive compared with their counterparts who had less frequent interactions with faculty. The study further reported that graduate students felt motivated and over time, developed a sense of belonging and professional identity if they were recognized as colleagues by faculty (Perna & Hudgins, 1996).

In this study, we found that the willingness of students to acquire professional knowledge and skills is a basis for socialization. Even though the ideals and expectations of individuals may differ, self‐motivation and enthusiasm to grasp the professional culture and values create opportunities for socialization to flourish. For these students, the way to achieve their learning goals is by developing professional relationships with supervisors, mentors, clients and peers. Much of student's knowledge acquisition is through role and skill observations, which often leads to the acquisition of vital professional skills. Other researchers have similarly reported the significance of student nurse role observations as being key to knowledge acquisition (Davies, 1993). Cope & colleagues opined that knowledge acquisition and competence development are essential elements in an individual's development of a sense of social acceptance (Cope, Cuthbertson, & Stoddart, 2000).

Self‐motivation is an essential element that gives students the pressure to succeed with a strength to pursue amidst challenges. Students have been reported to work hard in an attempt to demystify societal myths and to prove their worth. The eagerness and enthusiasm were identified as a contributor to their successful socialization. Likewise, we found that internal motivation is critical in role acceptance, which sets a basis for high confidence among students. These findings are similar to findings of previous studies which reported that nurses use personal resilience as a way to adjust positively to workplace adversities (Jackson, Firtko, & Edenborough, 2007).

Regarding educational facilitators, we found that on‐campus traditional nursing programmes that are structured to favour physical student interactions rather than online‐based programmes create perfect avenues for socialization to flourish. The students gain the opportunity to network, learn and socialize with others from different backgrounds and get to learn the hidden cultures of the profession.

### 6.1 Limitations

The main limitation of this study is that it focuses only on one subgroup of nurse trainees, that is undergraduate nursing students. Also, the limited number of studies included in this review will limit the generalization and practicality of some results presented. As a social phenomenon, the context where socialization occurs is critical. Therefore, areas whose studies are not featured in this review may present with different encounters. Hence, readers should interpret and apply the current findings with caution.

### 6.2 Relevance to clinical practice

Socialization is a critical aspect of nursing practice that aids in developing professional identities and nurturing professionalism among nurses. The findings of this review should serve as a guide to faculty members and clinicians in their quest to promote socialization among students and within practitioners. Sufficiently socialized students evolve to become competent professional nurses.

There is a need for further research in this area, especially so to identify specific challenges applicable to specific geographical context.

### 6.3 Recommendations

- Based on professional experience and cues from literature, we suggest the following recommendations to facilitate socialization among undergraduate nursing students.
- For socialization to be successful, a collaborative effort is required. Professional bodies, academy and individual professionals should get involved in dealing with the barriers of socialization.
- On successful admittance into nursing schools, students should be guided and assisted in selecting mentors.
- Orientation programmes should be organized to welcome and introduce newly admitted students to the profession.
- Student's concerns and perceptions should be addressed immediately.
- Professional nursing bodies must collaborate with the academy to identify and deal with barriers to socialization at academic and clinical centres.
- Courses should be designed to include some elements of socialization, and students should be encouraged to participate.
- Academic courses that favour minimal student interactions should be restructured to include some student activities that will ensure frequent interactions. It could be done through online platforms that will require students to participate mandatorily.
- Students should be encouraged to participate in professional events.
- Effective mentorship programmes should be established and encourage students to participate
- Nurse educators, mentors and clinicians should emphasize and encourage students to socialize professionally in the profession and venture to attain a minimum understanding, through socialization, how other related professions operate. Being a process lasting from studentship until graduation and beyond, socialization could be viewed as a lifelong learning opportunity for nurses.

## 7 CONCLUSION

Socialization in nursing contributes to moulding novice nurses and unveiling the professional cultures. However, the process is faced with barriers, presented in this review as challenges and situations that ease the process, presented herein as facilitators. Professional nursing bodies and academy are pivotal in achieving successful socialization processes. However, individual efforts are equally paramount.

## CONFLICT OF INTEREST

The authors declare that they have no competing interests.
